# Satellite RNAs and Satellite Viruses of Plants

**DOI:** 10.3390/v1031325

**Published:** 2009-12-18

**Authors:** Chung-Chi Hu, Yau-Heiu Hsu, Na-Sheng Lin

**Affiliations:** 1 Graduate Institute of Biotechnology, National Chung Hsing University, Taichung, 402, Taiwan; E-Mails: cchu@dragon.nchu.edu.tw (C.-C.H.); yhhsu@nchu.edu.tw (Y.-H.H.); 2 Institute of Plant and Microbial Biology, Academia Sinica, Taipei, 115, Taiwan

**Keywords:** satellite RNA, satellite virus, replication, RNA silencing, pathogenicity

## Abstract

The view that satellite RNAs (satRNAs) and satellite viruses are purely molecular parasites of their cognate helper viruses has changed. The molecular mechanisms underlying the synergistic and/or antagonistic interactions among satRNAs/satellite viruses, helper viruses, and host plants are beginning to be comprehended. This review aims to summarize the recent achievements in basic and practical research, with special emphasis on the involvement of RNA silencing mechanisms in the pathogenicity, population dynamics, and, possibly, the origin(s) of these subviral agents. With further research following current trends, the comprehensive understanding of satRNAs and satellite viruses could lead to new insights into the trilateral interactions among host plants, viruses, and satellites.

## 1. Introduction

Several viruses, as obligate parasites to the host plants, are associated with even smaller molecular parasites, sometimes being commensal or even beneficial, and are among the simplest life forms, namely, satellite RNAs (satRNAs) and satellite viruses. These satRNAs are short RNA molecules, usually <1,500 nt, that depend on cognate helper viruses for replication, encapsidation, movement, and transmission, but most share little or no sequence homology to the helper viruses [1]. In contrast, satellite viruses are satRNAs that encode and are encapsidated in their own capsid proteins (CPs). Certain satRNAs code for nonstructural proteins, but most satRNAs do not encode any functional protein products and are therefore thought to exert their biological functions through direct RNA interactions. Recent advances in research into satRNAs and satellite viruses have resulted in deeper insights into the molecular biology of these small replicating entities and to certain practical applications in modern biotechnology.

Satellite viruses and satRNAs have attracted much interest over the past decades, mainly for the following reasons [2–5]: (1) they can modulate − attenuate or exacerbate − the symptoms caused by their cognate helper viruses [6–8]; (2) they do not encode their own RNA-dependent RNA polymerases (RdRps) for their own replication and apparently use replication machineries similar to those of the helper viruses and thus have great potential as surrogate systems for the study of the replication mechanisms of their cognate helper viruses; (3) they can alter − usually reduce − the accumulation of their cognate helper viral RNAs and are thus considered the molecular parasites of the helper viruses; and (4) they can accumulate to high levels in host plants and thus in some cases can be developed into high-level expression vectors for foreign genes [9,10]. Because of these features, satRNAs and satellite viruses are good biological systems for the study of the molecular biology of viruses. Indeed, current studies of satRNAs and satellite viruses have provided further insights into several key subjects in molecular virology. In addition, recent studies have revealed the roles of an antiviral defense system of host plants, RNA silencing or posttranscriptional gene silencing (PTGS) in the pathogenicity and molecular biology of satRNAs. This information has altered our views of satRNAs and satellite viruses as being purely parasitic to the helper viruses (e.g., [5,11,12]).

This review focuses on current achievements in several important topics concerning satRNAs and satellite viruses, namely replication mechanisms, pathogenicity, and molecular evolution. Comprehensive information for all satRNAs and satellite viruses characterized previously has been provided in several reviews [1,3,5,13,14]. By understanding satRNAs and satellite viruses, we may gain further knowledge of the molecular biology of their cognate helper viruses and host plants, which might be useful in effectively managing viral diseases of plants and developing satRNA/satellite virus-based vector systems.

## 2. General classification of satRNAs and satellite viruses

**Figure 1.**
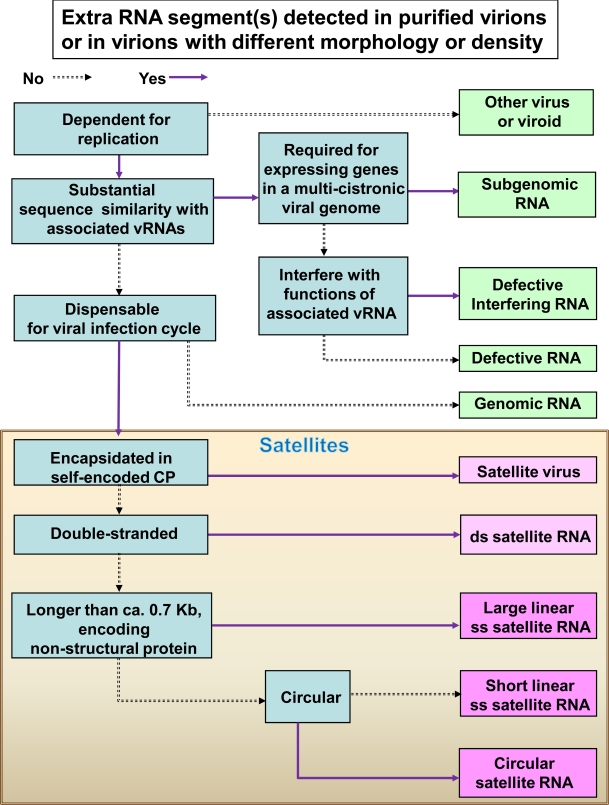
A key illustrating the hierarchical classification of subviral RNAs. Blue boxes describe salient features distinguishing different categories of subviral RNAs, pink boxes show satellite viruses and satRNAs, and green boxes show the other types of RNAs. Abbreviations: ss, single-stranded; ds, double-stranded; vRNA, viral genomic RNA; CP, coat protein.

To assist in distinguishing the different categories of sub-viral RNAs, an illustrated key is provided in Figure 1. Each category of subviral RNA can be identified according to the salient features listed in the key by the detection of additional RNAs from the purified virus particles (virions) or from virions with different morphology and/or density. The satellites, which are categorized at a status equivalence of “family” (NCBI Taxonomy ID: 12877), can be classified into the following types: single-stranded RNA satellite viruses, single-stranded satellite DNAs, double-stranded satRNAs, and single-stranded satRNAs [15,16]. Of note, satellites do not constitute a homogeneous taxonomic group [15] and are classified mainly according to the nature of the genetic materials. Satellite DNAs are usually circular DNAs of about 1.3 kb that encode a nonstructural protein known as βC1. The satellite DNAs, also known as DNAβ components, are associated with monopartite begomoviruses [15]. These DNAβ components play important roles in the pathogenicity of begomoviruses [e.g., 17].

Another shorter, noncoding form of satellite DNAs, of about 680 nt, has been found associated with *Tomato leaf curl virus* [18]. Furthermore, a nanovirus-like DNA component, termed DNA1, which encodes its own Rep protein, is also associated with begomovirus diseases [19]. The focus of this review is on subviral RNAs; thus in the following sections we restrict the discussion of satellites to members belonging to single-stranded RNA satellite viruses and single-stranded satRNAs associated with plant viruses. For further discussions of single-stranded satellite DNAs, please refer to the recent reviews [20,21].

By definition, all satellites share the following features: they depend on helper viruses, at least for replication; they are not part of the helper viral genome and are not required for the infection cycle of their helper viruses (with at least one exception: the satRNA associated with *Groundnut rosette virus*, GRV, [22]); and they share little or no nucleotide sequence similarity with their cognate helper viruses, which distinguishes them from subgenomic or defective RNAs (D-RNAs).

### 2.1. Single-Stranded RNA Satellite Viruses

There are two subgroups in this type [15,23]: subgroup 1, Chronic bee-paralysis virus-associated satellite virus*,* which contains a single member, Chronic bee-paralysis satellite virus [24]; and subgroup 2, Tobacco necrosis satellite viruses, which include the four members Maize white-line mosaic satellite virus [25], Panicum mosaic satellite virus [26], Tobacco mosaic satellite virus [27], and Tobacco necrosis satellite virus [28,29]. With respect to conventions, in the following section, these satellite viruses are referred to by names or acronyms traditionally used among plant virologists; for example, Panicum mosaic satellite virus is referred to as Satellite panicum mosaic virus (SPMV) in recent publications [30]. The four species of satellite viruses in subgroup 2 are associated with helper viruses in the genera *Aureusvirus, Panicovirus, Tobamovirus, and Necrovirus*. These satellite viruses share no sequence similarities with each other, which suggests that they might have originated from independent events in evolutionary history. Among them, only Satellite tobacco mosaic virus (STMV) has a rod-shaped virus as the helper [31]. Of note, deletion of nucleotides A and G at positions 1 and 61, respectively, of STMV, which is naturally adapted to *Tobacco mild green mosaic virus*, is required for adaptation to other helper viruses, namely *Tobacco mosaic virus* (TMV), *Tomato mosaic virus* or *Green tomato atypical mosaic virus* [32]. Satellite viruses can co-evolve with the helper viruses whenever they encounter each other. The recent advances concerning satellite viruses are discussed in section 4.

### 2.2. Single-stranded satRNAs

Single-stranded satRNAs are classified into three subgroups: subgroup 1, large satRNAs; subgroup 2, small linear satRNAs; and subgroup 3, circular satRNAs. Subgroup 1 contains large satRNAs of about 0.7 to 1.5 kb that encode at least one nonstructural protein. Thus, satRNAs in this subgroup are usually referred to as messenger-type satRNAs. Species in subgroup 1 recognized by the International Committee on Taxonomy of Viruses include Arabis mosaic virus large satRNA (satArMV), Bamboo mosaic virus satRNA (satBaMV), Chicory yellow mottle virus large satRNA, Grapevine Bulgarian latent virus satRNA, Grapevine fanleaf virus satRNA (satGFLV), Myrobalan latent ringspot virus satRNA, Strawberry latent ringspot virus satRNA, Tomato black ring virus (TBRV) satRNA, TBRV G serotype satRNA, and Beet ringspot virus satRNA [15,33]. Although the actual biological functions of many nonstructural proteins encoded by satRNAs are unknown, the protein encoded by TBRV satRNA has been detected *in vivo* and is involved in its replication [34]. The P3 protein encoded by a large satRNA, designated RNA3, of 1,114 nt, and associated with GFLV-F13, is also involved in satRNA replication [35]. In addition, the protein encoded by the satArMV is required for the *in planta* replication of the satRNA [36]. In contrast, the P20 protein encoded by satBaMV RNA [37] is not essential for replication but is involved in the systemic movement of satBaMV RNA [38,39]. It preferentially binds to 5′ and 3′ untranslated regions (UTRs) of satBaMV RNA [40] and interacts with the CP, a movement protein of its helper virus [triple gene block protein 1 (TGBp1)] and P20 itself [39]. The subcellular localization and expression kinetics of P20 have recently been investigated in detail [41], which further suggests its involvement in long-distance movement of satBaMV RNA. In addition to the satRNA-encoded proteins with reported functions, *Blackcurrant reversion virus* in the genus *Nepovirus* of the family *Comoviridae* also contains a satRNA, which is 1,432 nt and encodes a single protein of 402 amino acids and has unknown function [42].,

Species in subgroup 2 of single-stranded satRNAs include Cucumber mosaic virus (CMV) satRNA, Cymbidium ringspot virus (CymRSV) satRNA, Pea enation mosaic virus satRNA, GRV satRNA, Panicum mosaic virus small satRNA, Peanut stunt virus (PSV) satRNA, Turnip crinkle virus (TCV) satRNA, and Tomato bushy stunt virus (TBSV) satRNA B10, and TBSV B1 [15,33]. Tobacco necrosis virus small satRNA and Robinia mosaic virus satRNA are listed as tentative species in this subgroup. The satRNAs in this subgroup are short (usually less than 700 nt), linear RNA molecules that do not exhibit any biologically significant messenger activity. Among the short linear satRNAs, those associated with TCV, CMV, and PSV are the most thoroughly characterized (for reviews, see [3–5,43]). Strictly speaking, one of the satRNAs associated with TCV, namely satC, does not fit the definition of satRNAs, because satC is a hybrid molecule composed of a true satRNA, satD, and two fragments from the 3′ terminus of TCV genomic RNA [44]. However, studies of the satC have revealed many important factors and mechanisms involved in the infection cycle of both TCV and satC, which will be discussed in detail in the following sections.

Subgroup 3 includes the following species: ArMV small satRNA, Cereal yellow dwarf virus-RPV satRNA (previously Barley yellow dwarf virus satellite RNA), Chicory yellow mottle virus satRNA, Lucerne transient streak virus satRNA, Solanum nodiflorum mottle virus satRNA, Subterranean clover mottle virus satRNA (2 types), Tobacco ringspot virus satRNA, and Velvet tobacco mottle virus satRNA [15,33]. Species in this subgroup are characterized by a small (shorter than 400 nt), circular RNA genome without biologically significant messenger activity. These circular satRNAs, previously referred to as virusoids, replicate through a rolling-circle mechanism and self-cleave into monomers by use of endogenous hammerhead ribozyme activity (e.g., [45–47]).

## 3. Newly identified satRNAs or satellite viruses

Despite great technical advances in molecular virology, only a few new satRNA species have been identified over the past five years. These newly identified satRNAs are all associated with helper viruses in genera previously known to harbor satellites. These new satRNAs include the following. 1) A new satRNA was associated with the disease carrot motley dwarf (CMD), which is caused by a mixed infection of two viruses - the polerovirus *Carrot red leaf virus* and one of the umbraviruses, *Carrot mottle mimic virus* or *Carrot mottle virus* [48]. 2) A new satRNA associated with CMV emerged after serial passages of one strain of CMV, CMV-Fny, in *Nicotiana tabacum* cv. Ky 14, in an attempt to identify the origins of satRNAs [49]; no new satRNAs were observed in the same serial-passage experiment with the other strain, CMV-LS. Through nucleotide sequence analysis, the newly emerged satRNA was shown to be unique, which minimized the possibility of contamination. The observation is important because it indicates that satRNAs might be generated naturally during complex interactions among the invading viruses and host plants. 3) A novel satellite RNA associated with *Beet black scorch virus*, a necrovirus, was identified and molecularly characterized [50]; both mono- and dimeric forms of single- or double-stranded satRNAs were observed, which raised the possibility that the novel satRNAs replicate through multimeric intermediates.

The apparent lack of new satRNAs or satellite viruses identified in other new genera of plant viruses may reflect at least two scenarios: 1) satellites associated with other genera of plant viruses cause very mild symptoms or do not cause differences in symptoms inflicted by their cognate helpers because of long-term co-evolution and thus did not attract much research attention; or 2) viruses of the other genera have developed resistance through long-term co-evolution with satRNAs. Although previous studies have provided evidence for the existence of mutual beneficial relationships between satellites and helper viruses [5,22,51,52], most satellites may exert negative effects on their helper viruses, an observation that might eventually lead to the extinction of such satellite/helper-virus combinations. The latter scenario is further supported by the recent finding that CymRSV may direct the RNA silencing mechanism of the host plants to control the accumulation of the associated satRNA [12]. This observation may explain why satRNAs and satellite viruses are rarely observed in natural settings and why these satellites are found associated with only certain genera of viruses or certain species within the same genus of viruses.

## 4. Recent advances regarding satellite viruses

Several novel findings regarding SPMV in recent years have provided insights into how satellite viruses may cause symptoms. In addition to forming the protective shell for RNAs and facilitating systemic invasion, the CP of SPMV was demonstrated to elicit symptoms on a nonhost plant *Nicotiana benthamiana* [53]. When expressed by the *Potato virus X* (PVX)-based vector, the SPMV CP alone could cause necrosis symptoms on *N. benthamiana*. Omarov *et al.* [54] further demonstrated that in addition to the full-length, 17-kDa SPMV CP, a 9.4-kDa C-terminal protein, which exclusively co-fractionated with the cell wall- and membrane-enriched fractions, could also be translated from the third in-frame start codon of SPMV RNA. The expression of this 9.4-kDa C-terminal protein was linked to the severe symptoms caused by SPMV, possibly by interfering with the host membranes. As well, SPMV CP has multiple functions, including facilitating efficient satellite virus infection and movement in millet plants. In accordance with its multifunctional role, SPMV CP was recently found to be localized to cytoplasm, nucleus, cell membranes and cell walls. Although the biological significance of this complex subcellular distribution was not fully elucidated, the phenomenon suggests that SPMV CP is involved in both cell-to-cell transport and nuclear localization, which might affect the host-defense responses [30] and might help explain why SPMV CP could enhance the accumulation and the spreading speed of PMV in millets [55]. Most recently, the ability of SPMV CP to increase the stability of homologous and heterologous RNAs has been applied in the development of plant virus-based vectors [56]. SPMV CP expressed from a PVX-based vector could function *in trans* to protect the RNAs transcribed from a *Tomato bushy stunt virus*-based vector harboring a GFP gene and facilitate the systemic expression of GFP, further demonstrating the multifunctional nature of SPMV CP. On the other hand, the multiple biological activities of SPMV CP are regulated separately by the N-and C-terminal regions [57]. The results indicated that the CP of satellite viruses serves as the structural unit of virion assembly and has multiple biological functions involved in intra- and intercellular and long-distance movement. Thus, various protein products may be expressed from different initiation codons of the same open reading frame (ORF) on the satellite virus genome, and different subcellular localizations of the CP are possibly associated with multiple biological functions.

Considerable research advances have also been achieved for another well studied satellite virus system, the Satellite tobacco necrosis virus (STNV). The RNA of STNV does not contain a cap structure and poly(A) tail at the 5′- and 3′-termini, which is required for efficient translational initiation by interacting with eIF4E and poly(A)-binding protein, respectively. However, the RNA of STNV can still be efficiently translated by use of the translational enhancer domain (TED), which locates within the 122-nt region in the 3′ untranslated region of STNV RNA, to recruit the translational machinery [58,59]. Thus, STNV provides a useful system for understanding the molecular interactions between RNA structures and translation factors during the translational initiation events.

Use of the STNV system to study the early translational processes revealed that the first 38 nt at the 5′-terminal region of STNV RNA could function synergistically with the 3′ TED to enhance the translational efficiency [60]. Later, the 3′-TED was revealed to interact with either of two protein factors, p28 or p30, in the wheat germ *in vitro* translation system to recruit translation machinery [61]. The boarder sequences of the 3′-TED were further identified through the analyses of the translational stimulation effects in wheat germ extracts (*in vitro*) and tobacco protoplasts (*in vivo*) using serial deletion mutants as templates [62]. The results revealed that STNV TED might contain at least two functional domains that might interact with different translational factors. Van Lipzig *et al.* [62] established an alternative secondary structure model of 3′-TED involving the base-pairing of functional sequences of the 5′- and 3′-termini. This model elegantly shows the presence of two possible domains with different translational stimulation effect *in vitro* and *in vivo* and supports the possibility that RNAs might adopt different structures for different functions, e.g., replication and translation. In addition, STNV TED was later shown to specifically interact with the eukaryotic initiation factors eIF4E and eIF(iso)4E [63]. Interestingly, the genomic RNA of the helper virus TNV was found to contain a *Barley yellow dwarf virus*-like cap-independent translation element (TE) at the 3′-terminal region. Although all known members of the genus *Necrovirus* contain similar TE structures, the TE structure was not present in STNV [64], which suggests that the helper viruses and the satellites might have different evolutionary origins or might have adopted different strategies for survival.

## 5. Recent advances regarding satRNAs

Research on satRNAs has been a very active area in virology and has led to great insights into the molecular biology of their helper viruses and host plants. Recent advances include the following.

### 5.1. Replication mechanisms

As mentioned previously, satRNAs may serve as model systems for the study of biological functions, namely replication and translation. As the non-messenger-type satRNA, satC, associated with TCV, is among the best-characterized satRNAs, both for structure and function. Although satRNAs depend on the replicases of the cognate helper viruses for replication, the required sequence or structural elements were not always found to be functionally interchangeable between the satRNAs and cognate helper viruses [65,66]. Despite the high similar sequence and structure in the 3′-terminal 150 nt of both RNAs, the 3′- terminal regions of satC and TCV are not fully interchangeable. The accumulation of recombinant satC or TCV containing reciprocal exchanges of the 3′-terminal regions was greatly reduced to only 15% or 1%, respectively, that of the wild types [67]. The results suggest a dependence on cognate promoter regions for efficient RNA accumulation. Recent studies have revealed that the 3′ terminal structures of both TCV and satC are dynamic, being able to switch between structures for translation or replication depending on the interaction of RdRp in the case of TCV [68] or between the pre-active and active structures for replication of satC [66,69]. The observations reflect the difference in the requirement for functions: satC is a non-messenger-type RNA that does not need the presence of translational signals. The structural plasticity and the rapid evolutionary adaptation to certain structures with high “fitness” by satC variants were further demonstrated by *in vivo* genetic selection experiments (SELEX) [70]. Furthermore, a 3′ proximal translational enhancer in TCV genomic RNA was able to bind to 60S ribosomal subunits [71]. The results support that the access of RdRp to multiple elements, including the 3′ end of TCV, causes structural changes that potentiate the shift between translation and replication [68]. Together, these results convey the concept that viral and satellite RNA conformations may undergo switches by the regulation of viral or host factors depending on required biological functions [for a thorough review, see 72].

The notion that satRNAs may adopt different replication signals and use replicase complexes different from those used by their helper viruses is also true for the messenger-type satRNAs. Annamalai *et al.* [73] and Huang *et al.* [74] analyzed the structural and sequence requirements on the 5′- and 3′-UTRs of satBaMV RNA, which encodes a non-structural protein, P20, by enzymatic probing and mutational studies, respectively. The evolutionarily conserved secondary structures, as well as certain nucleotides, in both the 5′- and 3′-UTRs were found essential for the efficient replication of satBaMV RNAs. Huang *et al.* [74] further demonstrated partial similarities in structures and functions in the 3′-UTRs of BaMV and satBaMV RNA. However, differences between the 3′-UTRs of BaMV and satBaMV RNA were identified: 1) satBaMV RNA does not contain the 3′-terminal pseudoknot structure, and replacing the 3′-terminal portion of satBaMV with the pseudoknot structure of BaMV had a lethal effect on satBaMV RNA; 2) the requirement for the hexanucleotides, ACCUAA [75,76], conserved in all known potexviruses, in the loop region of stem-loop C (SLC) of satBaMV is not as strict for satBaMV RNA; and 3) regardless of the high degree of structural similarity, the 5′- and 3′-UTRs of BaMV and satBaMV RNA were not interchangeable. These results suggest that satRNAs might have adopted a 3′ UTR with structural features similar to but distinct from those of the cognate helper viruses for their own efficient replication.

Comparable phenomena were observed for satellite viruses. Rubino *et al.* [77] demonstrated that co-expressing the replication proteins of *Carnation Italian ringspot virus* (CIRV) in yeast cells could support the replication of several defective interfering RNAs derived from the genome of CIRV or the related CymRSV but not that of a satRNA originally associated with CymRSV. Taken together, the results suggest that satRNAs have adopted a system similar to but distinct from their cognate helper viruses for their replication and thus are not strong competitors of their helper viruses. This suggestion may explain why satRNAs share only limited or no sequence similarities to their cognate helper viruses.

### 5.2. RNA silencing mechanisms of the host plants are involved in the pathogenicity of satRNAs and their co-evolution with their helper viruses

Three types of symptom modulation are associated with satRNAs: attenuation, exacerbation, and no significant effect. Symptom attenuation is the most commonly observed effect. In most plant species, most satRNAs reduce the replication and accumulation of their helper viruses, which results in the amelioration of symptoms. In some cases, the satRNAs that attenuate the symptoms in several host plants may cause exacerbated symptoms in certain other hosts, such as lethal necrosis in tomato [6] and strong systemic chlorosis in tobacco, pepper, and tomato [78–80].

Accumulating evidence in recent years supports that RNA-silencing mechanisms of the host plants play important roles in the pathogenicity and evolution of satRNAs. RNA silencing, also referred to as PTGS or RNA interference (RNAi), is a double-stranded, RNA-induced, sequence-specific RNA degradation process, considered one of the important anti-viral mechanisms of plants [81,82]. To counter the defense system of host plants, RNA viruses usually encode proteins that function as suppressors of RNA silencing. The CP of TCV suppresses RNA silencing in *N. benthamiana* by obstructing the Dicer-like protein DCL2/DCL4 silencing pathway [52,83,84]. TCV CP is a weak suppressor of RNA silencing when assembled into the intact virions but a strong suppressor when expressed freely in the cytosol. The satC associated with TCV can reduce the accumulation of virions, thereby increasing the level of free CP, which leads to the suppression of RNA silencing and subsequent exacerbation in symptom severity [5,51]. Thus satC may actually contribute to the fitness of TCV. In another virus/subviral RNA system, Wang *et al.* [11] showed that the RNA silencing mechanism is involved in the pathogenicity of satRNA and viroids. The transgenic expression of a potent silencing suppressor of a potyvirus, helper component-protease (HC-Pro), in *N. tabacum*, greatly attenuated the severe yellowing symptoms caused by the Y satellite of CMV. The expression of self-complementary hairpin RNAs derived from the Potato spindle tuber viroid (PSTVd) in tomato plants resulted in symptoms similar to PSTVd infection. These results provide strong evidence that an RNA silencing mechanism is involved in the pathogenicity of satRNAs, whereby satRNAs direct RNA silencing against biologically important host genes. Furthermore, Wang *et al.* [11] demonstrated that viroid and satRNAs are significantly resistant to RNA silencing-mediated RNA degradation, which indicates that RNA silencing may be the driving force for the evolution of these subviral RNAs.

#### 5.2.1. The pathogenicity of satRNAs is determined by a complex competition among host plants, helper viruses, and satRNAs in the targetting of the RNA silencing mechanism

In the past, the pathogenicity of satRNAs has been thought to be a direct interaction among the satRNAs, helper viruses and host factors by an unknown mechanism(s). Taking cucumoviruses and associated satRNAs as examples, a satRNA of CMV was originally reported to be the causal agent of lethal necrosis disease of tomato in France [6]. Since then, many satRNAs with different symptom modulation effects have been reported. The pathogenic effects of cucumovirus satRNAs were suggested to result from a trilateral interaction among the host plant, the helper virus strain, and specific sequences of the satRNA [85–92]. Most PSV satRNAs do not have an apparent effect on their helper viruses or host plants. However, one satRNA of PSV, G-sat, was found to suppress viral RNA replication in noninoculated leaves of PSV-ER–infected tobacco plants [93]. At that time, no information was available on the involvement of RNA silencing pathways in satRNA pathogenicity, yet a hint of the interaction of specific regions of satRNA, helper viral RNA or host factors was apparent.

The determination of nucleotide sequences of a large number of biologically distinct satRNAs has facilitated the identification of specific sequences responsible for differential modulation of symptoms. For example, by inoculating tobacco plants with mutant forms of satCMV, Jaegle *et al.* [87] demonstrated that the yellow mosaic symptom determinant of the satCMV Y strain lies in nucleotide position 185/186. In addition, early studies of several laboratories have mapped the necrogenic ability of satCMV RNAs on tomato to a 15-nt region near the 3′ termini [94–97]. For PSV systems, a U and a C residue at nucleotides 226 and 362, respectively, contribute to the symptom attenuation of PSV G-sat RNA [98]. However, the focus of these previous researches was mainly on the nucleotide sequences as the symptom determinants of satRNAs. It is now becoming clearer that host factors or mechanisms are the key determinants of the pathogenicity of viruses and satRNAs. Indeed, the involvement of ethylene in the induced defense responses has been recently demonstrated in a large-scale transcriptome study analyzing systemic programmed cell death caused by CMV and associated D satRNA as a model system [99]. With the increasing number of plant genomes being completely sequenced [100], it is expected that more host genes or regulatory sequences involved in symptom development will be revealed soon.

In the aforementioned scenarios (section 5.2.), the plant antiviral defense systems have been turned against the plants themselves by the invading virus/satRNA combinations. In response, plants have adapted mechanisms of defense. For example, one of the key proteins in the antiviral RNA silencing pathway, DCL4, can target the satRNAs of CMV at novel secondary structures, preferably T-shaped hairpins, instead of the classic double-stranded hairpins, to produce small interfering RNAs (siRNAs) against satRNAs [101]. The results suggest that RNAs of diverse structures can be potent stimuli to activate antiviral RNA silencing mechanisms in plants.

Furthermore, helper viruses may also exploit the RNA silencing mechanisms of host plants to control the accumulation of parasitic satRNAs. The accumulation of a parasitic satRNA of CymRSV strongly depends on the presence of the RNA silencing suppressor P19 of the helper virus, which suggests the involvement of an RNA silencing mechanism in the competition of CymRSV and satRNA. As well, siRNAs derived from CymRSV targeted satRNA with higher efficiency than did the siRNA derived from a distantly related helper virus, CIRV [12]. Thus, CymRSV apparently had adapted well to the host plant so that it could harness the RNA silencing activity of the host plants against satRNA to control its accumulation. Many classic examples exist of satRNAs reducing the concentration of the helper viruses and thereby attenuating the symptoms elicited by the helper viruses. Recently, satRNA was found to possibly cause the reduction in accumulation of the genomic RNAs of CMV-Fny in *N. tabacum* [102]. The reduction in accumulation of helper viral RNAs was possibly related to the 2b gene of CMV, encoding a silencing suppressor, which suggests the involvement of an RNA silencing mechanism. Furthermore, Hsu *et al.* [103] and Chen *et al.* [104] reported that the specific conserved 5′ structure, an apical hairpin stem-loop (AHSL), and sequences in the internal loops of satBaMV RNA play an essential role in downregulating the expression of helper viral RNAs. The conserved AHSL structure could be used as a potent determinant of the ability to downregulate the expression of BaMV. Although the underlying mechanism remains to be elucidated, the results support satRNAs modulating the pathogenicity of the helper viruses through the specific structures and sequences in the untranslated regions, possibly by tweaking the RNA silencing pathways of the hosts. Obviously, RNA silencing mechanisms of host plants are among the key determinants of the pathogenicity of satRNAs and helper viruses. However, further studies are required to identify the actual target(s) of RNA silencing that result in different types of symptoms.

#### 5.2.2. A simplified model illustrating trilateral competition for the targeting of RNA silencing mechanisms

**Figure 2.**
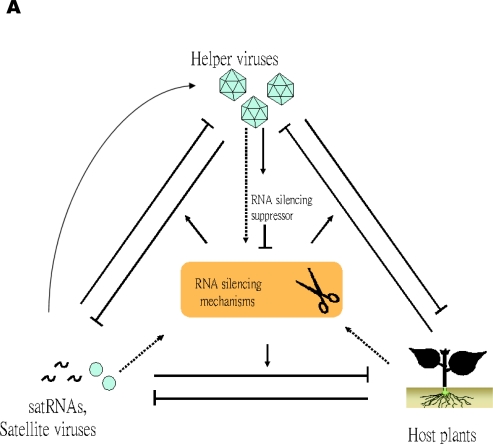

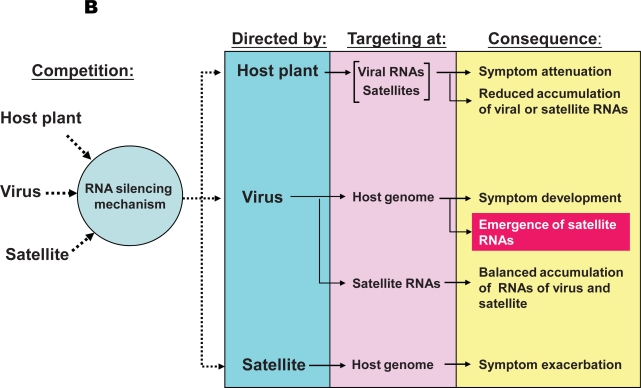
**A** Schematic representation of the complex interaction among host plants, helper viruses, satRNAs and satellite viruses, and the RNA silencing mechanism of the host. T-shaped lines indicate inhibitory effect, whereas the solid arrows represent an enhancing effect. Arrows on dotted lines represent an “uncertainty state,” in which the three participating members (host plants, helper viruses, and satRNAs or satellite viruses) would have to compete for the targeting of host gene silencing mechanisms. **B** Example scenarios illustrating the consequences of competition among plants, viruses, and satellites for the activation and targeting of the RNA silencing mechanisms. The yellow box indicates the emergence of new satRNAs as a result of an RNA silencing mechanism.

Recent studies indicating the involvement of RNA silencing pathways in the pathogenicity of satRNAs have provided new insights into the underlying mechanisms of satRNA-mediated symptom modulations. Clearly, the pathogenicity of satRNAs is the result of a complex interaction among satRNAs, helper viruses, RNA silencing mechanisms, and the physiologically important genes of the host plants (Figure 2 A), which could be regarded as a competition for the use, or targeting, of the RNA silencing systems of the host plants. Example scenarios depicting the consequences of the trilateral competition are in Figure 2 B. If the host plants activate the RNA silencing system against the invading virus and satRNAs, the system can be considered an antiviral and anti-satRNA defense system. However, if the RNA silencing systems are programmed by satRNAs against mRNAs of host plants, the system can be considered as using the pathogenic factors of the satRNA to target the mRNAs of the physiologically important gene for degradation. In rare cases (e.g., [51,52]), the satRNAs may assist the helper viruses (the curved arrow in Figure 2 A) in counter-defending the RNA silencing pathways in plants. In contrast, viruses may express proteins as suppressors of RNA silencing (for reviews, see [105–108]) to inhibit the innate defense systems of host plants and elicit symptoms or even “harness” the RNA silencing mechanism against satRNAs to downregulate their expression, as was shown for CymRSV and the associated satRNA [12].

### 5.3. Possible Origin(s) of satRNAs

A long-standing question regarding satRNAs is the origin of these small RNAs. Considering the infection cycle of satRNAs, at least three natural sources are the origin of satRNA segments: the genomes of the helper viruses or other co-infecting viruses/satellites, the host organisms, and transmission vectors. Although many reports claimed the “spontaneous” occurrence of satRNAs under experimental conditions, previous attempts to locate the sequences with significant similarity to satRNA in the genomes of hosts, helper viruses and vectors have in general been unsuccessful [109,110]. However, one cannot rule out that the source sequences of satRNAs exist as separate fragments in these genomes but, after being transcribed, can be recognized by the replicase complexes of the invading viruses and reassemble into functional RNA molecules. These molecules are further selected under various host or environment conditions to emerge as satRNAs [5].

Previous research found that 3′-terminal deletions of an satRNA of TCV, satD, could be repaired *in vivo* [111]. The satD transcripts with different 3′-terminal deletions were repaired to either a wild-type satD sequence or to other recombinant forms containing additional deletions joined to an internal TCV genomic RNA (or host) sequence, then replacement of the terminal motif [112]. The authors presented an elegant repair mechanism involving primer-mediated abortive synthesis of oligoribonucleotides by the viral RdRp, with internal regions of TCV genomic RNA used as templates, then the recombinational repair of the 3′-terminal deletions of satD. The results suggested that satD may have evolved by the repeated recombination of the TCV genomic RNA-derived oligoribonucleotides generated by TCV RdRp in these abortive synthesis events, then replication and encapsidation by TCV. In addition, Nagy *et al.* [113] extended the mechanism of 3′-end repair to another satRNA, satC. With TCV viral RNA used as a template, the authors demonstrated that TCV replicase could synthesize a pool of oligoribonucleotides that could be used as primers for the subsequent RNA synthesis in repairing the damaged 3′ ends of satC. Together, these results support that satRNAs might have originated from the genomes of the helper viruses.

In the natural plantation, sometimes more than one species of viruses/satellites may infect the same plant. Thus, the genomes of other co-infecting viruses or satellites have also been suggested to be another possible origin of satRNAs. For example, the sequence of the nonstructural protein P20 of satBaMV was found to share 46% identity with the CP of SPMV and could be folded into a similar “jelly-roll” structure [114,115]. On the basis of sequence similarity, satBaMV RNA and SPMV might share a common evolutionary origin.

The genome of *Arabidopsis thaliana* contains stretches of nucleotide sequences with significant similarity to satCMVs [5]. The possibility that host genomes might serve as the sources of satRNAs is further supported by the recent identification of many siRNAs or microRNAs (miRNAs) generated or transcribed by various mechanisms in plants [116,117]. These small regulatory RNA molecules might be produced in response to viral infections and could possibly be assembled into satRNAs by viral replicases. Furthermore, the genomes or extra-chromosomal genetic materials of other microorganisms residing inside the host cells might be sources of recombination that eventually lead to the generation of satRNAs. Accordingly, sequence segments with significant similarities to satRNAs could be identified in the genomes of hosts and the microorganisms, and the constituents of the satRNA population could differ in various hosts from diverse geographical distributions. Modern advances in whole-genome sequencing projects and bioinformatics techniques have yielded great resources of data and tools to test these hypotheses.

Recently, a novel satRNA of CMV was reported to have emerged naturally following serial passages of CMV-Fny [49]. Great efforts were made to eliminate the possible sources of contamination. The finding raised the intriguing possibility that the novel satRNA may have been generated *de novo* from the genomes of CMV and host plants after the activation of RNA silencing mechanisms, which would generate a large quantity of small RNA fragments for assembly into the initial form of satRNAs. That satRNAs were not detected when CMV-LS was used for serial passages suggested the main involvement of viral genomes in the emergence of satRNAs. Although the experimental conditions did not completely eliminate the possibility of contamination, as with performing the whole experiments in a new laboratory environment never exposed to satRNAs [5], as acknowledged by the authors [49], this work pointed to the importance of the influence of invading viruses on host innate immunity, the RNA silencing pathway, as an alternative underlying mechanism for the generation of satRNAs [5,52].

In view of the above notion, the sequence similarities between satRNAs and viruses or host plants may reflect the genes targeted in the host or viral genomes by use of siRNAs derived from satRNAs. As mentioned previously, satRNAs and viroids may direct the RNA silencing mechanism against biologically important genes of the cognate host plants [11]. Similarly, as illustrated in Figure 2, viruses may also harness the RNA silencing mechanism against satRNA [12] or important host genes. The resulting small RNA fragments may be the origins of satRNAs in these systems. As predicted by the Red Queen Hypothesis [118], following the initial generation of fragments, the small RNA fragments may recombine and evolve rapidly to adapt to the replication and encapsidation systems of the helper viruses. The involvement of RNA silencing pathways in the generation of satRNAs may also be used to explain the intriguing phenomenon that satRNAs were mostly associated with plant hosts but rarely found in animal systems. In vertebrates, the active immune system involving antibodies may play major roles in defense against invading viruses, with the RNA silencing pathways serving as an innate defense system. Thus, it is less likely that siRNAs could be generated in large amounts to be recombined and evolved to the forms that could be replicated and encapsidated by the helper viruses.

### 5.4. Molecular evolution: constraints on the structures of satRNAs; linear comparison of multiple secondary structures

Because most satRNAs lack any significant coding capacity, they are expected to express their biological functions through direct interaction of the structure or nucleotide sequences of satRNAs with the respective host factors. Thus, the structures of satRNAs are under the constraint of their biological functions in evolutionary history. In addition, the conservation of certain structural elements may contribute to the stability and/or efficient accumulation of satRNAs.

**Figure 3.**
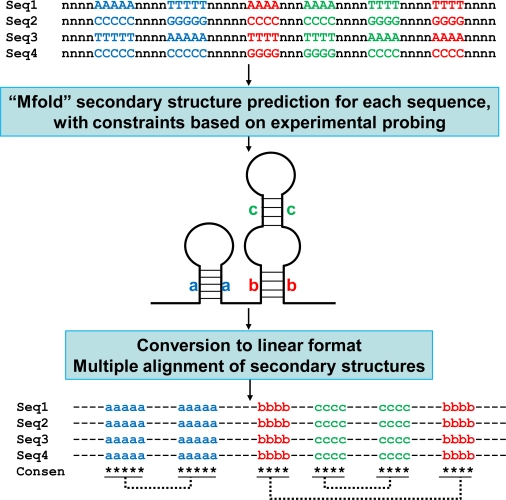
Identification of conserved secondary structures among sequences sharing low similarity of satRNAs. The flow chart outlines the process for the linear comparison of multiple secondary structures. Only the relevant nucleotides participating in base-pairing are indicated. Nucleotides in single-stranded regions are represented by the letter “n”. For each sequence (Seq1–Seq4), secondary structures were predicted by the program Mfold [119], with constraints matching the structures determined by enzymatic or chemical probing experiments. The resulting connect file (“.ct file”) was converted to a linear format according to the rules [74] and subjected to multiple sequence alignment by use of Clustal W [120]. The base-paired regions are represented by letters in lower case, a, b, and c, with dotted lines connecting the corresponding regions. The conserved base-paired regions among different sequences are indicated by stars under the alignment. Consen, consensus secondary structure derived from the multiple alignments.

SatRNAs and viroids have evolved and maintained secondary structures that are considerably more resistant to RNA silencing-mediated degradation [11]. Sun and Simon [121] provided evidence to support that satC associated with TCV has evolved a core 3′-terminal promoter structure that is more efficient for RdRp binding than that of TCV. Several recent studies also revealed the crucial role of specific secondary structures of satBaMV RNAs in symptom determination and downregulation of the helper virus BaMV [73,103,104]. In addition, Huang *et al.* [74] performed structural and functional analyses of the 3′-UTR region of satBaMV RNA and revealed that satRNAs may have evolved a functionally similar but structurally distinct promoter element for efficient replication. This type of work requires comparing and contrasting the secondary structures of many satRNA molecules. However, use of the current 2-D graphical output for each satRNA does not allow for presenting many secondary structures in a small space (e.g., on one page) for easy viewing. Huang *et al.* [74] developed a simple method to convert many secondary structures into a linear format to easily identify highly conserved structural features among the different RNAs. The conceptual explanation of the process is given in Figure 3. By using this method, two stem-loop structures at the 3′-UTR (SLA and SLB) were found identical among more than 60 natural occurring satBaMV RNA variants. Another stem-loop structure, SLC, was also highly conserved although not identical among satBaMV RNAs. The observation supported that SLA, SLB, SLC play important roles in the survival of satBaMV RNA. The linear comparison of multiple secondary structures facilitated the easy identification of conserved structures among many sequence variants [74]. These results reinforced the importance of the conservation of certain structural elements in the fitness of satRNAs..

## 6. Practical applications of satRNAs

Apart from the contribution of satRNAs in basic research into molecular virology, satRNAs have also been used in application-oriented studies. One of their potential applications lies in the development of satRNA-based vector systems for the expression of foreign genes in plants. In comparison with using other types of vectors for foreign gene expression, such as plasmids and viral vectors, using satRNAs and satellite viruses as vectors has the following advantages: i) ease of manipulation, ii) high *in vivo* stability, and iii) high expression level. First, because of the relatively small size of satRNAs and satellite viruses, they are simpler systems to use for cloning, sequencing, genetic modification, and regular maintenance. Second, most satRNAs are highly structured and thus significantly more resistant to degradation by nucleases *in vivo* than are other viral RNA-based vector systems. For instance, about 49% of the genome of one of the most thoroughly characterized satCMVs participates in the formation of base-paired structures [122], which increases the robustness of the satRNA *in planta*. And finally, most satRNAs and satellite viruses can accumulate to high levels in host plants, which leads to the increase in level of proteins translated from the messenger-type RNAs of the satellites. However, despite the aforementioned potentials, most attempts to develop vectors based on coding or non-coding satRNAs for applications in biotechnology have failed, probably because of the strict requirement for the maintenance of certain secondary structures, which severely limited the sequence alterations that could be introduced. The current applications of satRNAs are demonstrated only for the following rare cases:

1. As vectors for *in planta* expression of foreign genes:
  The practical application of satBaMV RNA was demonstrated more than ten years ago [38], and remains the sole example of such applications. Being a messenger-type satRNA, satBaMV RNA encodes a P20 gene, which, unlike all other large messenger-type satRNAs, was shown to be nonessential for the replication of satRNA. Therefore, P20 was replaced with a chloramphenicol acetyltransferase (CAT) gene, which resulted in high expression of the CAT protein in infected *Chenopodium quinoa* and demonstrated that satBaMV RNA could be useful as a satellite-based expression vector.
2. As vectors for functional studies:
  satBaMV RNA has been used for the expression of individual TGBps in a complementary experiment for study of cell-to-cell movement of BaMV [123]. To dissect the functions of individual TGBps, single or multiple TGBps of BaMV, PVX and *Foxtail mosaic virus* were expressed by use of satBaMV RNA-based vectors to complement the functions of green fluorescent protein-tagged, movement-defective BaMV with mutation(s) in the matching gene(s). The results revealed the requirement for species-specific interactions among TGBps for cell-to-cell movement of BaMV and possibly other potexviruses. In addition, a satBaMV-based vector system has been used in analysis of promoter sequences to generate potexvirus subgenomic RNAs (sgRNAs) [124]. Insertion of subgenomic promoter-like sequences (SGPs) into the upstream of the start codon of the P20 gene gave rise to the synthesis of sgRNA of satBaMV in infected cells co-inoculated with helper BaMV RNA. By deletion and mutational analyses, one core promoter-like sequence, two upstream enhancers and one downstream enhancer were identified in the function of SGP *in vivo*. Such applications in functional analysis further demonstrated the additional advantages of satRNA-based vectors: i) Applicable for mutational studies of essential genes or regulatory sequences: satRNAs are physically separated from the viral genomes and are not essential for the infection cycle of the helper viruses; thus, they can be used in mutational or complementary assays of regulatory sequences. ii) Convenient for combinational studies on multiple genes: two or more satRNA-based vectors harboring different genes may co-exist in one plant and be supported by the same helper virus, although they might be not expressed at the same level.
3. As vectors for gene silencing:
  Gossele and Metzlaff [9] described the use of STMV vectors for gene silencing. The work was an extension of their previous work with a satellite virus-induced silencing system (SVISS) [125]. The authors demonstrated the potential of STMV as a vector of gene silencing by knocking out (or knocking down) the expression in *N. tabacum* of a variety of genes involved in different biochemical pathways (e.g., phytoene desaturase, glutamine synthetase, chalcone synthase, and transketolase). Most of the silenced phenotypes could be observed 10–12 days postinoculation, which indicates the efficiency of STMV-based vectors in the induction of gene silencing [125].

In addition, the symptom-attenuation features of satRNAs have been successfully exploited in developing satRNA-based disease-management systems, either directly, with satRNAs used as biological control agents [2,126], or indirectly, by producing transgenic plants that express satRNA sequences [127–129]. The occurrence of some virus strains that do not support the replication of satRNAs in certain host plants may present an obstacle to satRNA-mediated disease management. However, satRNAs still provide us with experimental tools for understanding the complex trilateral interaction among satRNAs, helper viruses, and host plants.

## 7. Concluding Remarks

With significant advances in research into satRNAs and satellite viruses, our understanding of the pathogenicity, evolution, and molecular biology of satRNAs and satellite viruses is now gaining momentum. Further breakthroughs can be expected soon. Although the detailed mechanisms remain to be elucidated, clearly, RNA silencing mechanisms are involved in these processes: satRNAs may use RNA silencing mechanisms of the host plants to target biologically important genes to exacerbate symptoms [11], and viruses may harness similar RNA silencing mechanisms to target parasitic satRNAs to suppress their accumulation [12]. The sequence-specific nature of the RNA silencing mechanism may help explain why a minute alteration in the nucleotide sequences or the combination of satRNAs, helper viruses, or host plants could lead to significant differences in the outcome of interactions, causing attenuation or exacerbation of or even no effect on the accumulation and symptom expression of satRNAs and helper viruses. Even the origins of the satRNAs or satellite viruses may involve the RNA silencing mechanism, as was suggested by Simon *et al.* [5]. The authors proposed that satRNAs might have originated from small regulatory RNAs such as siRNAs and miRNAs generated from RNA silencing mechanisms induced by the infection of viruses.

Considerable progress has been made in the comprehensive understanding of satRNAs and satellite viruses; however, several intriguing questions still remain. The following topics, for example, those concerning the involvement of RNA silencing mechanisms in the pathogenicity, population dynamics, and possibly the origin(s) of satRNAs and satellite viruses, are expected to continue to attract the attention of researchers in the coming years. (1) How do viruses, satellite viruses, or satRNAs actually “harness” the RNA silencing mechanism of their host plants for their own benefits? (2) What are the actual targets of RNA silencing mechanisms in host genomes directed by satRNAs and satellite viruses that eventually lead to the development of symptoms? (3) Given that satRNAs depend on helper viruses for major biological functions, why do certain satRNAs observed in laboratories or specific field conditions evolve to compete with or reduce the expression of their helper viruses, instead of evolving toward a mutually beneficial situation, as has been observed for TCV and satC [5,49]? Clearly, further experiments are needed to resolve these issues. The resulting knowledge will shed light on the molecular biology of satRNAs and satellite viruses, and provide insights into that of the cognate helper viruses and host plants.
